# *Pasteurella multocida* Epiglottitis

**DOI:** 10.5811/cpcem.2016.11.32294

**Published:** 2017-01-18

**Authors:** Andrey Moyko, Nissa J. Ali, Nicole M. Dubosh, Matthew L. Wong

**Affiliations:** Beth Israel Deaconess Medical Center, Department of Emergency Medicine, Boston, Massachusetts

## Abstract

Epiglottitis is an uncommon but life-threatening disease. While the most common infectious causes are the typical respiratory pathogens, *Pasteurella multocida* is a rare causative organism. We present a case of *P. multocida* epiglottitis diagnosed by blood culture. The patient required intubation but was successfully treated medically. *P. multocida* is a rare cause of epiglottitis; this is the ninth reported case in the literature. Most diagnoses are made from blood culture and patients usually have an exposure to animals.

## INTRODUCTION

Epiglottitis is characterized by inflammation of the epiglottis and surrounding structures and classically presents with severe sore throat, dysphagia, and leukocytosis.^1^ Although uncommon, it is a life-threatening disease that can cause acute airway obstruction. The most common etiology of epiglottitis is an infection by *Streptococcus pneumonia*, *Staphylococcus aureus,* or *Haemophilus influenzae* (type B, A, F, and nontypable), while other bacteria, viruses, and fungi constitute more rare forms.^2^,^3^,^17^–^19^

*Pasteurella multocida* is a Gram-negative coccobacilus that colonizes the respiratory system of dogs and cats, as well as commercial livestock and wild animals.^4^ In cows *P. multocida* is a leading cause of bacterial pneumonia and morbidity known as bovine respiratory disease.^10^ In humans, there are only eight reported cases of *P. multocida* epiglottitis in the literature since 1977.^1^,^2^, ^4^–^9^ All eight reports involve middle-aged adults, seven of whom were men. Three cases involved immunosuppressed patients.^1^,^4^,^5^ Seven cases involved contact with animals, generally domesticated cats.^1^,^2^,^3^–^7^,^9^ Pasteurella was diagnosed as the causative agent by blood culture in seven cases, with the remaining case by post-mortem tissue culture.^1^,^2^,^4^–^9^ We present a case of *P. multocida* epiglottitis involving a healthy middle-aged woman who had two pet dogs but could not recall any bites, scratches, or inoculations.

## CASE REPORT

A 49-year-old female with a past medical history of hypothyroidism, prurigo nodularis, and depression presented to our emergency department (ED) with one day of gradual onset of sore throat, dysphagia, odynophagia, and chills. She was very concerned because she had the sensation that she could not swallow her own saliva. On exam she was non-toxic appearing, afebrile, and had normal vital signs. Her voice and oropharyngeal exam were normal. Her external neck was notable for mild left submandibular lymphadenopathy. The rest of her exam was normal. She did not reliably take her levothyroxine at home for her hypothyroidism, she had no known allergies, no known sick contacts, no recent travel, nor did she use illicit drugs; her immunizations were up to date.

She was given a dose of ketorolac and observed. On reevaluation she was unable to tolerate any liquids by mouth. Her oropharynx and larynx were then topicalized with lidocaine and a flexible fiberoptic endoscope was inserted orally to evaluate the larynx. The patient had a beefy, red, and edematous epiglottis with edema and erythema extending to the aryepiglottic folds and arytenoids, and she had mild pooling of secretions. The true vocal folds were symmetric and mobile without erythema or edema. An image from the initial laryngoscopy can be seen in Image 1. The patient was administered dexamethasone, ampicillin-sulbactam, diphenhydramine, and racemic epinephrine nebulizers. Her complete blood count was notable for a white blood cell count of 22.6 cells per microliter with a 91% neutrophil predominance, and two sets of blood cultures were taken. The otorhinolaryngologist we consulted performed a nasopharyngolaryngoscopy and recommended medical management in the intensive care unit (ICU).

Several hours after admission to the medical ICU the patient’s respiratory efforts increased, and she was taken to the operating room where the anesthesiologist performed an awake nasotracheal fiberoptic intubation with the acute care surgery team at the bedside for a potential emergent surgical airway. The patient was successfully intubated nasotracheally and returned to the ICU. The blood cultures drawn in the ED returned positive for pleomorphic Gram-negative rods on the first hospital day, which were later speciated for pan-sensitive *P. multocida*. A viral respiratory panel from the nasopharynx returned negative for adenovirus, respiratory syncytial virus, parainfluenza virus types 1, 2, and 3, as well as influenza viruses A and B.

The patient had a midline catheter placed for home antibiotic infusions, and her antibiotics were tailored to ceftriaxone monotherapy. On hospital day three, laryngoscopy showed much improvement of her epiglottitis and the patient was successfully extubated. She was transferred to the medical-surgical floor on hospital day four, and then discharged home on hospital day eight with a plan for continued antibiotic infusion. During her hospitalization the infectious disease (ID) service was consulted for treatment recommendations. The ID team recorded a history of the patient owning a Chihuahua and a terrier at her home, though she could not recall any recent scratches, bites, or contact with animal saliva or mucous membranes. The patient followed up in otorhinolaryngology clinic one month later and had a repeat laryngoscopy, which did not show any evidence of epiglottitis.

## DISCUSSION

Epiglottitis caused by *P. multocida* is rare; only eight other cases have been reported in the literature since 1977.^1^, ^2^, ^4^–^9^ *P. multocida* is a Gram-negative coccobacillus commonly found in the upper respiratory tracts of animals, including domestic dogs and cats.^4^ Exposure to animals was a common theme in previously reported cases of epiglottitis secondary to *P. multocida*. Patients with immunosuppression may have a greater susceptibility to infection.^1^,^11^ Other risk factors for epiglottitis include lack of immunization and smoking.^11^ *Haemophilus influenza* type B (*HiB*) used to be the most prevalent organism, but its incidence declined dramatically after the introduction of the pediatric vaccination series. Currently group A beta-hemolytic streptococci is the most common causative bacterial organism, and pediatric epiglottitis is less common than adult-onset epiglottitis.^12^

Our patient was an otherwise healthy 49-year-old woman without any significant co-morbidities that would suggest immunosuppression. She did have regular exposure to her two dogs, and it is possible that oral secretions from her dogs were the source of the infection; however, the exact mechanism of exposure was not confirmed.

The clinical presentation of adult patients with epiglottitis most commonly involves the rapid onset of sore throat with dysphagia, odynophagia and the inability to swallow secretions. 1,13 A normal oropharyngeal exam does not exclude epiglottitis. Additional signs may include a tripod patient positioning, cervical lymphadenopathy, and fever.^13^, ^14^ Laboratory results often show a leukocytosis.^1^ The causative organism can be confirmed with epiglottic tissue and blood cultures.^1^ Treatment with penicillins, cephalosphorins, or fluoroquinolones is recommended for 1–6 weeks, depending on the severity of infection.^1^,^5^ Steroids, racemic epinephrine and beta-agonists may also be considered, although they have not been proven to improve outcomes.^13^

Epiglottitis is suggested on lateral neck radiographs that demonstrate a “thumb sign,” which indicates increased epiglottic and aryepiglotticwidth.^13^,^15^ The epiglottis is typically 3–5 mm thick on a lateral neck radiograph.^13^ The “vallecula sign” may also be seen on the lateral radiograph, in which a V-shaped space is observed extending from the base of the tongue to the epiglottis instead of a normal linear space.^13^ However, one study showed that lateral radiography only had a sensitivity of 38% and specificity of 76% for diagnosing epiglottitis.^16^ An ultrasound of the neck can also demonstrate an increased anteroposterior diameter of the epiglottis in epiglottitis and may aid in diagnosis.^17^ Computed tomography is very accurate at diagnosing epiglottitis.^21^ Suspected epiglottitis is probably best evaluated by direct visualization of the epiglottis.

Airway management is the cornerstone of therapy for severe cases and milder cases with poor trajectory. Intubating a patient with epiglottitis (or suspected epiglottitis) should be presumed to be very challenging, and an awake intubation with simultaneous preparation for a possible surgical airway is likely the most prudent option; a surgical airway may be inevitable. The airway may need to be managed in the ED, but transferring the patient to the operating room with anesthesia and surgical backup may be more prudent.

Emergency physicians should be aware of this diagnosis because epiglottitis is a life-threatening disease. *P. multocida* epiglottitis is rarely the pathogen responsible, though diagnosis can be made by blood culture. Epiglottitis is rare in adulthood, but sore throat, dysphagia and odynophagia are common chief complaints. If a patient reports that they cannot tolerate their own secretions, further investigation is warranted.

## Figures and Tables

**Image:**
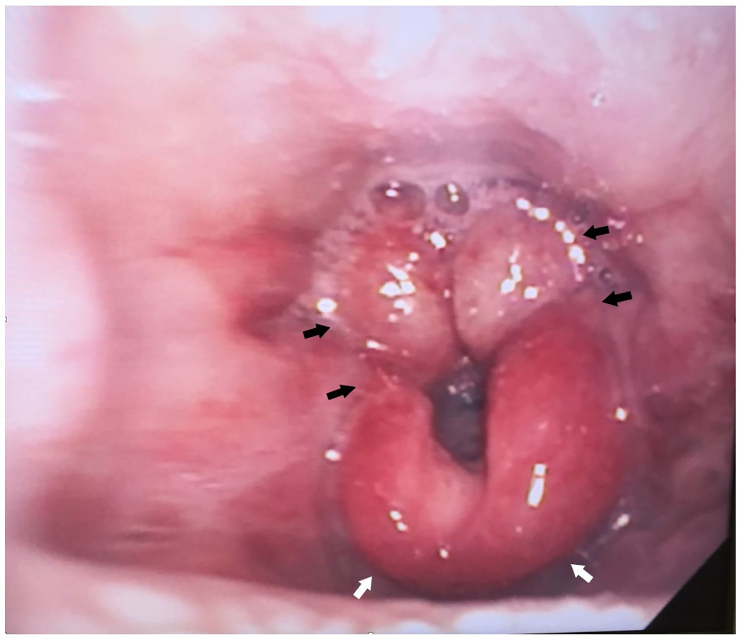
Emergency department endoscopy demonstrating epiglottitis (white arrows), swelling of the aryepiglottic structures (black arrows), and pooling of secretions.
